# Fibrillin microfibrils and elastic fibre proteins: Functional interactions and extracellular regulation of growth factors

**DOI:** 10.1016/j.semcdb.2018.07.016

**Published:** 2019-05

**Authors:** Jennifer Thomson, Mukti Singh, Alexander Eckersley, Stuart A. Cain, Michael J. Sherratt, Clair Baldock

**Affiliations:** a Wellcome Trust Centre for Cell-Matrix Research, School of Biological Sciences, Faculty of Biology, Medicine and Health, University of Manchester, Manchester Academic Health Science Centre, Manchester, M13 9PT, UK; b Division of Cell-Matrix Biology and Regenerative Medicine, School of Biological Sciences, Faculty of Biology, Medicine and Health, University of Manchester, Manchester Academic Health Science Centre, Manchester, M13 9PT, UK

**Keywords:** Fibrillin, Elastin, Fibulin, LTBP, ADAMTS

## Abstract

Fibrillin microfibrils are extensible polymers that endow connective tissues with long-range elasticity and have widespread distributions in both elastic and non-elastic tissues. They act as a template for elastin deposition during elastic fibre formation and are essential for maintaining the integrity of tissues such as blood vessels, lung, skin and ocular ligaments. A reduction in fibrillin is seen in tissues in vascular ageing, chronic obstructive pulmonary disease, skin ageing and UV induced skin damage, and age-related vision deterioration. Most mutations in fibrillin cause Marfan syndrome, a genetic disease characterised by overgrowth of the long bones and other skeletal abnormalities with cardiovascular and eye defects. However, mutations in fibrillin and fibrillin-binding proteins can also cause short-stature pathologies. All of these diseases have been linked to dysregulated growth factor signalling which forms a major functional role for fibrillin.

## 1 Introduction

Fibrillin, an extracellular matrix glycoprotein, assembles into microfibrils, a component of many connective tissues, where they form the template for elastic fibre formation. Fibrillin is also found in tissues devoid of elastin such as the ciliary zonules of the eye. In elastic fibre assembly, it is understood that elastin globules are deposited directly onto a microfibril template and the elastic fibre is comprised of an amorphous elastin central core surrounded by a fibrillin microfibril sheath [1]. A wide array of fibrillin binding proteins are known to facilitate the assembly of elastic fibres and contribute to their functionality, and these will be discussed herein.

## 2 Fibrillin microfibrils

Fibrillin microfibrils, which have a beads-on-a-string appearance, are a major component of elastic fibres, conferring long range extensibility and contributing to the elastic deformation of tissues. Microfibrils also play a key role in tissue homeostasis through their interaction with growth factors such as transforming growth factor-β (TGFβ) and bone morphogenetic proteins (BMPs) and through interaction with cell surface receptors such as the integrins [2,3] and syndecans [4]. The importance of fibrillin-1 in the function of tissues is further highlighted by fibrillin-1 mutations that cause a number of heritable connective tissue disorders termed fibrillinopathies, such as Marfan syndrome (MFS) [5], Weill Marchesani syndrome (WMS) [6] and geleophysic dysplasia (GD) [7].

**Fig. 1:**
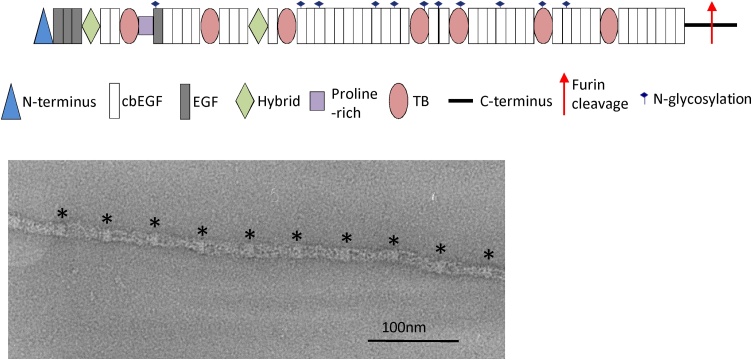
The domain organisation of fibrillin-1 is shown. Fibrillin assembles into microfibrils with a beads on a string appearance. A microfibril is imaged by negative stain TEM and the beaded structure is highlighted by asterisks.

Though three fibrillin isoforms have been identified in humans, fibrillin-1 (FBN1) is most abundant in adult tissues. Fibrillin-1 is a glycoprotein composed of 2871 amino acids with a molecular mass of ∼320 kDa [1]. It consists of 59 domains including an N-terminal region, epidermal growth factor-like repeats (EGF), most of which are calcium binding (cb), TB (TGFβ-binding like) domains and hybrid domains which bear homology to both cbEGF domains and TB domains (Fig. 1). It has been suggested that arrays of domains have a linear, rod-like structure, modelled from high resolution structures of domain pairs and triplets [8,9]. However, X-ray scattering data show that, in solution, longer domain arrays from fibrillin and the homologous latent TGFβ binding protein (LTBP)-1 are flexible and adopt non-linear conformations [10,11]. Fibrillin assembles into microfibrils, but despite knowing the structures of some domains, how individual fibrillin monomers are arranged in the microfibril is still not understood. Fibrillin microfibrils imaged in tissues have a diameter of ∼10-12 nm [12] with a 57 nm periodic beaded structure [13] and in cross-section they appear hollow with a ring of eight filaments [14,15]. They have a mass per repeat ranging from ∼1400 kDa, in some early foetal tissues and cell culture, to ∼2500 kDa in adult tissues [16]. This mass is consistent with up to eight fibrillin monomers per repeat [13].

## 3 Fibrillin-binding proteins

**Fig. 2:**
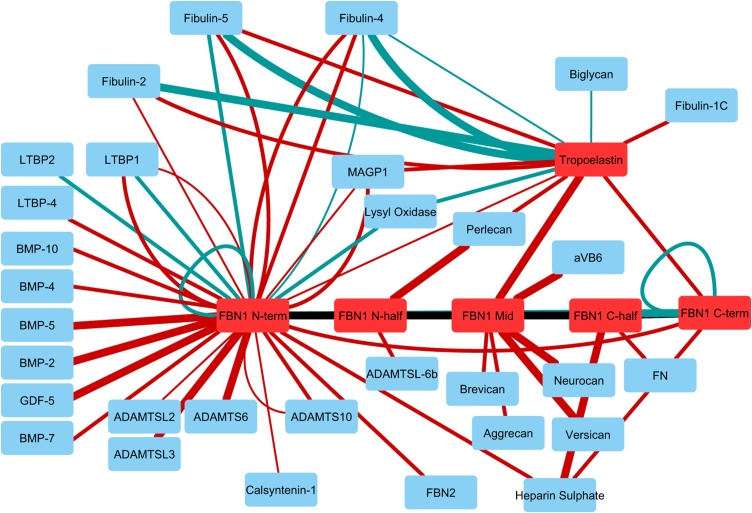
An interaction network showing the fibrillin and tropoelastin interactions with their binding proteins listed in Table 1. The line width increases with increasing interaction strength (from >100, 10–100 to 1–10 nM Kd) and the line colour indicates either SPR (red) or solid phase binding (blue).

A large number of molecules have been shown to colocalise or interact with fibrillin. Here we have focussed on key binding proteins that have been shown to bind directly with fibrillin or tropoelastin, the soluble elastin precursor, and have roles in microfibril assembly or growth factor regulation. Table 1 shows the direct molecular interactions between fibrillin and tropoelastin that have been quantified and these are also presented in Fig. 2.

**Table 1:** Affinity between fibrillin-1 or tropoelastin and their binding proteins with the protein interaction analysis approach (SPR = surface plasmon resonance, IP = immunoprecipitation, Solid Phase = solid phase binding assay) and dissociation constant (Kd).

Protein A	Protein B	Reference	Approach	Kd (nM)
FBN1 N-ter	ADAMTS10	[17,18,19]	SPR	11-35; 245
FBN1 N-ter	ADAMTS6	[18]	SPR	1-7
FBN1 N-ter	ADAMTSL2	[19]	SPR	200
FBN1 N-ter	ADAMTSL3	[19]	SPR	6
FBN1 N-half	ADAMTSL5	[20]	IP	N/A
FBN1 N-half	ADAMTSL6β	[21]	SPR	80
FBN1 N-half	Aggrecan	[22]	SPR	49 and 42
FBN1 cbEGF22-TB4-cbEGF23	Integrin αVβ6	[3]	SPR	1
FBN1 N-ter	BMP-2, 4, 5, 7, 10, GDF5	[23,24]	SPR	6 - 34
FBN1 N-ter	Brevican, Neurocan, Versican	[22]	SPR	20, 2, 7.1
FBN1 N-ter	Calsyntenin-1	[25]	SPR	240
FBN1 N- and C-ter	FBN1	[26]	Solid Phase	5-11
FBN1 N-ter	FBN1 C-ter	[27]	SPR	3-25
FBN1 N-ter	FBN2 C-ter	[27]	SPR	10-74
FBN1 N-ter	Fibulin-2	[28]	SPR	160
FBN1 N-ter	Fibulin-4	[28,29]	SPR	74; 54
FBN1 N-ter	Fibulin-5	[28,29]	Solid Phase, SPR	63; 23
FBN1 C-half	Fibronectin	[30]	SPR	55
FBN1 N- and C-ter	Heparan Sulphate	[30,31]	SPR	27, 93; 16
FBN1 N-ter	LTBP1	[28]	SPR	21
FBN1 N-ter	LTBP2	[32]	Solid Phase	22
FBN1 N-ter	LTBP-4	[28]	SPR	24
FBN1 N-ter	Lysyl Oxidase	[29]	Solid Phase	26
FBN1 N-ter	MAGP1	[31,33]	SPR, Solid Phase	140-240; 36.5
FBN1 N-half	Perlecan	[34]	SPR	6-9
FBN1 N-ter	MFAP4	[35]	SPR	N.D.
Tropoelastin	Biglycan	[36]	Solid Phase	195
Tropoelastin	FBN1 N-ter, Mid, C-ter	[31,33]	SPR	280, 5; 27
Tropoelastin	Fibulin-1C	[37]	SPR	18
Tropoelastin	Fibulin-2	[38]	Solid Phase	1-2
Tropoelastin	Fibulin-2	[37]	SPR	18
Tropoelastin	Fibulin-4	[29,38]	Solid Phase	8, 131
Tropoelastin	Fibulin-5	[29,38]	Solid Phase; SPR	2; 64
Tropoelastin	Lysyl Oxidase	[29]	Solid Phase	49
Tropoelastin	MAGP1	[33]	SPR	22
Tropoelastin	Perlecan	[37]	SPR	21

### 3.1 Latent TGFβ binding proteins

Latent TGFβ binding proteins (LTBPs) 1–4 are extracellular glycoproteins with structural similarities to fibrillins, with long or short splice forms (L and S). LTBPs -1, -3 and -4 can covalently bind to the latency-associated peptide of TGFβ, producing the large latent complex (LLC) [39,40]. The LLC becomes sequestered within the matrix, and can regulate TGFβ bioavailability [[39], [40], [41]]. Both LTBP-1 L knockout and complete null mice (i.e LTBP-1S and L) die at birth from severe aortopathy [42,43]; the phenotype produced is similar to that observed in TGFβ-knockout aortic smooth muscle cells [44], supporting the requirement of LTBP-1 L for TGFβ signalling. LTBP-2 binds to the matrix via fibrillin [45,46] and, through binding to fibulin-5, negatively regulates elastic fibre assembly [47,48]. Mutations cause ectopia lentis (EL) [49,50] (a manifestation of MFS) and WMS and WMS-like syndrome [51] and loss of LTBP-2 in patients leads to congenital glaucoma [52,53]. LTBP-2 has also been implicated in wound healing [54]. LTBP-3 deletion in humans produces similar phenotypes to those of LTBP-3 null mice, including short stature, spinal curvature, craniofacial abnormalities and increased bone mass [[55], [56], [57]]. Aberrant LTBP-3-TGFβ complexes may be responsible for aortic dilation and dissection in MFS as MFS mice lacking LTBP-3 had fewer aneurysms and less fragmented elastic fibres [58]. LTBP-4 is required for proper elastogenesis, involving fibulin-4 and -5 incorporation into the matrix [[59], [60], [61], [62]]. Humans with inactive LTBP-4 develop autosomal recessive cutis laxa type 1C (ARCL1C), characterised by severe craniofacial, developmental and potentially fatal pulmonary defects [63]. Knockout studies using both LTBP-4S-/- and/or LTBP-4L-/- mice have uncovered discrete roles for these isoforms. Whereas mice that express only LTBP-4 L normally survive to adulthood [64], complete null mice develop an ARCL1C-like phenotype where mice die perinatally with highly impaired elastogenesis [60], likely due to lack of fibulin-4 in the matrix [61]. LTBP-4 has also been identified as a modifier of muscular dystrophy (MD) [[65], [66], [67], [68]]. MD mice with an LTBP-4 allele that contains an insertion in the hinge region had a less severe phenotype, including increased muscle mass and reduced fibrosis [65,68], coinciding with lower levels of extracellular TGFβ.

### 3.2 Fibulins

Fibulin-4 and -5 are extracellular glycoproteins that have roles in elastogenesis [38,69]. They bind to both tropoelastin and lysyl oxidase (LOX) or LOX-like 1 (LOXL1) [29,70,71] as well as to fibrillin-1 [29], thereby facilitating elastin cross-linking by LOX/LOXL1 and deposition onto microfibrils. Mutations in fibulin-4 (*EFEMP2*) cause ARCL1B, characterised by severe cardiovascular abnormalities, joint laxity and arachnodactyly [72]. LTBP-4S-/- mice with additional fibulin-4 deficiency have more perturbed elastogenesis [60,61], and fibulin-4 -/- mice develop significant lung and vascular defects and die perinatally [69], potentially due to altered LOX-mediated cross-linking and matrix interactions [73]. However, this dependency on fibulin-4 may vary with vessel type [74]. Mice with a human fibulin-4 mutation (E57 K) have disrupted elastic fibres and developed ascending aortic aneurysms [74,75], whereas muscular arteries and resistance vessels, such as those in the kidney and mesentery, have normal elastic fibres and cross-linking is unaffected [74]. Upregulation of fibulin-4 (and colocalisation with LOX1) has been observed in exfoliation syndrome, an elastic fibre disorder that leads to glaucoma [76] and bronchopulmonary dysplasia (BPD) [77]. Fibulin-5 depletion disrupts elastic fibre assembly to a lesser extent than fibulin-4 [78,79]. Patients with fibulin-5 (*DANCE*) mutations develop ARCL1A, characterised by loose skin, emphysema and arterial tortuosity. These mutations produce conformational changes that lower fibulin-5 secretion and affinity for tropoelastin [[80], [81], [82], [83], [84]].

### 3.3 Microfibril associated glycoproteins

Microfibril associated glycoproteins (MAGPs)-1 and -2 (also known as microfibrillar associated proteins (MFAPs)-2 and -5, respectively) copurify and colocalise with microfibrils [[85], [86], [87], [88], [89]]. Although neither is required for elastic fibre assembly [[90], [91], [92]], both may promote elastin deposition onto microfibrils [33,87,93]. However, MAGP-1 has been shown to influence wound healing, ciliary zonule formation, ageing, bone remodelling and thrombus formation [90,91,[94], [95], [96], [97]]. Despite the apparent lack of cardiovascular effects observed in either MAGP-1 or MAGP-2 knockout mice, depletion of both genes resulted in aortic dilation in older mice, suggesting a possible compensation mechanism [92]. Additionally, Barbier et al discovered an association between MAGP-2 mutations and thoracic aortic aneurysms and dissection (TAAD) [98]. Although little is known about the role of MFAP-4 in elastogenesis, Pilecki et al identified tropoelastin, fibrillin-1 and -2 and desmosine as MFAP-4 interaction partners in vitro. MFAP-4 also enhanced elastin assembly and colocalised with fibrillin-1 microfibrils in vivo [35].

### 3.4 ADAMTS and ADAMTSL proteins

A Disintegrin And Metalloprotease with Thrombospondin type-1 repeats (ADAMTS) and ADAMTS-Like (ADAMTSL) are matrix glycoproteins with many biological functions including morphogenesis, development, angiogenesis, inflammation and coagulation, as well as maintaining the structural integrity of tissues [99]. Recently, a subset of ADAMTS and ADAMTSL proteins have been implicated in microfibril assembly, adhesion and matrix stability [100]. Mutations in ADAMTS6 and -10 give rise to WMS [101]; gene disruption of ADAMTS17 leads to WMS-like syndrome [102]; and mutations in ADAMTSL2 and –L4 result in GD [103] and EL, respectively [104]. The association of ADAMTSs and ADAMTSLs with fibrillin-1 related pathologies suggests that they modulate fibrillin-1 function. Indeed, perturbations in the expression of ADAMTSs and ADAMTSLs disrupt the deposition of fibrillin microfibrils and TGFβ regulation [17,102,103]. Mouse models either lacking *ADAMTSL2* or bearing a nonsense mutation in ADAMTSL4 mimic phenotypic characteristics observed in patients with GD or EL, further corroborating the involvement of ADAMTSs and ADAMTSLs in these fibrillinopathies [[105], [106], [107]].

ADAMTS10 is involved in the biogenesis and maintenance of fibrillin-1 microfibrils, whereas ADAMTS6 inhibits microfibril deposition [17,18]. ADAMTS10 binds the N- and C-terminal regions of fibrillin-1 [17,19], while ADAMTS6 also interacts with the N-terminus of fibrillin-1 and the C-terminus of LTBP1 [18]. ADAMTS10 is required for and can enhance the formation of focal adhesion complexes through interactions with fibronectin and heparan sulphate (HS), whereas ADAMTS6 depletes HS and hence focal adhesions [18]. ADAMTS10 has a negative effect on ADAMTS6 expression, whereas ADAMTS6 shows synergistic effects on ADAMTS10 expression [18]. ADAMTS10 colocalises with fibrillin and addition of exogenous ADAMTS10 enhances microfibril deposition [17]. Mutations in ADAMTS17 also result in the dislocation of the ocular lens due to progressive degradation of the ciliary zonules in WMS-like patients [102], implying ADAMTS17 is also an accessory to fibrillin microfibril biogenesis and regulation.

ADAMTSL2 binds to the N- and C-terminus of fibrillin-1 [7,19], and the N-terminal binding site overlaps with a three domain fibrillin-1 deletion that causes WMS [19]. Increased levels of fibrillin-2 but not fibrillin-1 were observed in ADAMTSL2-deficient mouse lung [107]. This study also showed enhanced staining of LTBP-1 in bronchial tissues, increased levels of active TGFβ, as well as substantial epithelial dysplasia. ADAMTSL2 interacts with different regions of LTBP-1 suggesting that ADAMTSL2 may also play a role in regulating TGFβ availability in the matrix [19,103]. Found in ocular tissue, ADAMTSL4 is deposited in the matrix in a fibrillar arrangement co-localised with fibrillin microfibrils and addition of ADAMTSL4 to cultured fibroblasts enhances microfibril deposition [108,109]. Furthermore, analysis of ciliary zonules in mutant mice bearing a nonsense mutation in ADAMTSL4 revealed disorganised arrangements of fibrillin microfibrils [106] supporting a role for ADAMTSL4 in microfibril deposition.

### 3.5 Potential new associated proteins

Mass spectrometry has proven useful for identifying new fibrillin microfibril-associated proteins. MMP3 and annexins V and II co-purified with fibrillin microfibrils purified from human ciliary zonules [86]. Molecular fishing identified elastic fibre-associated proteins including fibronectin, perlecan, LOX, fibrillin-2, and TGFβ2 [25]. Again, the annexins were detected along with other candidate proteins, such as vimentin, βig-H3, thrombospondin-1, S100-A7, plasminogen activator inhibitor 1 (PAI-1) and IGF-binding proteins (IGFBP)-3 and -7 [25]. More recent characterisation of potential associated proteins from purified human ciliary body and skin fibrillin microfibrils identified MFAP5, versican and fibrillin-2 in both eye and skin-derived samples, whereas perlecan was identified solely in eye-derived samples and elastin, EMILIN-2 and fibulin-2 and -1 were identified solely in skin-derived samples [110]. Concordant with the molecular fishing study, annexins V and II, vimentin and βig-H3 all co-purified with eye and skin fibrillin microfibrils, whereas IGFBP7 and PAI-1 co-purified with only those derived from eyes. Interestingly the chaperones, protein disulphide isomerase and calreticulin, which play a role in intracellular fibrillin assembly [111], were also identified in both tissues (Table 2).

**Table 2:** Fibrillin-associated candidate proteins co-identification by molecular fishing and native tissue co-purification [25,110].

New Associated Protein Candidates	Known extracellular matrix interactions
Annexins V, II	Ca^2+^ channels, major components of matrix vesicles with activity stimulated by matrix binding e.g. collagens II and X [112].
Vimentin	Intracellular intermediate filaments interact with matrix indirectly via vimentin-associated matrix adhesions (VAMs) [113].
βig-H3	Matrix molecule with versatile roles in tissue homeostasis; interacts with numerous matrix components [114].
IGFBP3, -7	Modulate IGF in tissue which can be affected by their direct interaction with fibronectin [115].
PAI-1	Protease inhibitor mediates the degradation of matrix [116].

## 4 Functional modifiers of elastic fibres

### 4.1 Transglutaminase

Transglutaminases regulate matrix remodelling and are associated with numerous pathologies including cancer, inflammation and fibrosis [117]. Tissue transglutaminase is known to have a significant role in elastic fibre assembly, both in the cross-linking of fibrillin microfibrils [118] and between fibrillin-1 and tropoelastin [33,119]. The LLC and LTBP-1 N-terminus are also transglutaminase substrates [120], and LTBP-1 forms multimers (both N-N and N-C) that may be cross-linked [11], enhancing its incorporation into the matrix [121] and consequently, regulation of TGFβ signalling. Since proper incorporation of the latent complex is required for normal TGFβ regulation, this finding has implications for fibrillinopathies such as MFS, where these processes may become dysregulated when aberrant complexes are formed. Abnormal transglutaminase activity is also associated with BPD in premature infants and impaired mitral valve development, both attributed to a dysregulated matrix [122,123].

### 4.2 Lysyl oxidases

Lysyl oxidases LOX and LOX-like1 promote the cross-linking of elastin and its subsequent deposition onto microfibril scaffolds to form elastic fibres [124]. Consequently, LOX is essential for cardiovascular development and function, indeed null mice have defective arterial wall structure with fragmented elastic fibres and die perinatally from aortic aneurysms [125]. Lysyl oxidases may protect against aortic aneurysm formation in MFS, as both LOX and LOXL1 were expressed more highly in a MFS mouse model and in aortic tissue from MFS patients. In the mouse model, LOX inhibition prevented collagen deposition and exacerbated elastic fibre defects, leading to aggressive aortic dilation and death [126]. Guo et al recently identified the LOX mutation that causes familial TAAD: the Ser280Arg mutation produced a less active variant that led to disorganised elastic fibres and increased collagen in patients’ aortas [127]. Several reports have described an association between LOXL1 and glaucoma, specifically in the early stages of the elastic fibre disorder exfoliation syndrome (XFS) [76,[128], [129], [130], [131]]. LOXL1 colocalised with elastin, fibrillin-1 and fibulin-4, which were also upregulated, and its expression was enhanced by TGFβ1 and other XFS-associated stimuli in Tenon’s capsule fibroblasts [76].

## 5 Extracellular regulation of growth factor signalling

### 5.1 Transforming growth factor (TGF)β

As mentioned, dysregulated TGFβ signalling is a defining characteristic of MFS and other fibrillinopathies. Several studies have reported a direct association between elevated TGFβ signalling and MFS pathology in mouse models, where injection of a TGFβ-neutralising antibody restored alveolarisation and atrioventricular valve integrity and prevented aortic aneurysms, the most severe clinical feature of MFS [[132], [133], [134]]. However, there are a number of compelling reports that mitigate TGFβ’s role in disease pathology [135,136], while some point to angiotensin II as the major factor [[137], [138], [139]]. Some also argue a protective role for TGFβ [140,141]. Whereas TGFβ neutralisation reduced the incidence of aortic aneurysms in the study by Habashi et al [134], recent data suggests otherwise and, in some cases, show enhanced aneurysm formation upon TGFβ antagonism [140,141]. Neutralising TGFβ in smooth muscle cells increased aortic aneurysms in an AngII-induced model, but not when the antibody was administered systemically [136]. In order to directly address whether excess TGFβ signalling is responsible for the aortopathy observed in MFS, the same group deleted the type II TGFβ receptor in the smooth muscle cells of fibrillin-1 deficient mice. Aortopathy was exacerbated upon loss of TGFβ signalling, and was still observed even without changes to signalling in young mice [135].

A number of clinical trials support this emerging hypothesis. A trial using Losartan to block or diminish the effect of excess TGFβ appeared successful in reducing aortic dilation in MFS patients [142]. However, the same group later reported that Losartan efficacy depended on the patients’ fibrillin-1 status: patients with haploinsufficiency (i.e. a lower quantity of normal fibrillin-1) responded to Losartan better than those with dominant negative fibrillin-1 mutations (i.e. defective fibrillin-1) [137]. In a separate study, the authors determined that patient responsiveness to Losartan correlated with higher baseline levels of TGFβ and a higher aortic dilation rate; they concluded that TGFβ could be considered a biomarker for MFS status (as did Radonic et al [143]), but that angiotensin II triggers initial aortic dilation and increases TGFβ levels [138]. In support of these data, Losartan was no more effective in trials comparing it with other MFS therapies. For example, in the largest study of its kind, aortic dilation was not significantly different with Losartan than standard therapy with beta-blockers [139,144].

### 5.2 Bone morphogenetic proteins (BMP) and growth and differentiation factors (GDF)

MFS patients and fibrillin-1 insufficient mice have reduced bone mass, suggesting perturbed BMP signalling. Fibrillin-2 null mice have reduced bone formation and impaired osteoblast maturation, implicating a direct role for microfibrils in bone formation [145]. Moreover, abnormal activation of BMP signalling causes myopathy in fibrillin-2 null mice but deletion of a single allele of BMP7 rescues the muscle phenotype [146]. A direct interaction between BMP and the related GDF growth factors and fibrillin-1 was shown; BMP7 is secreted with its bound prodomain which binds fibrillin-1 [147]. Furthermore, GDF5, BMP2, BMP4 and BMP10 but not GDF8 prodomains bind fibrillin-1 via the N-terminal region showing widespread interaction of BMP/GDF family members with fibrillin-1 [23]. Indeed binding to fibrillin-1 was shown to induce latency for proBMP7 where a conformational change from the activatable open form to the closed latent form was observed upon fibrillin-1 binding [148]. A role for BMP antagonists in modulating extracellular regulation of BMPs via fibrillin was shown for gremlin-1. Fibrillin-2 is upregulated in mesothelioma and localises gremlin-1 to the tumour microenvironment where it binds fibrillin-1 and -2 and colocalises with microfibrils in cells and mesothelioma tumours [149].

## 6 Summary and future directions

Fibrillin microfibrils are periodic, multi-component assemblies with a widespread tissue distribution. In most organs they associate with elastin to form elastic fibres and hence make key contributions to tissue mechanics and structure function. Although there are three fibrillin isoforms, in adults fibrillin-1 is most abundant. In tissues it forms calcium-stabilised microfibrils which are thought to be composed of eight monomers per repeat. In addition to elastin, fibrillin microfibrils interact and/or co-localise with proteins from the ADAMTS/L, LTBP and fibulin families and with proteoglycans and enzymes. The LTBPs and fibulins both share considerable structural homology with the fibrillins as all three families are characterised by multiple cbEGF domains. These proteins play key roles in elastogenesis and mutations in LTBP and fibulin genes, in common with Marfan syndrome causing fibrillin-1 mutations, can manifest as pathologies in multiple organ systems. The pathological mechanisms conferred by LTBP mutations are driven by dysregulation of TGFβ signalling whilst fibulin mutations may induce aberrant tissue remodelling as a consequence of impaired LOX interactions. In contrast the role of MAGPs in elastogenesis is less well defined but knockout experiments and MAGP genetic mutations are clearly associated with pathologies in elastin-rich tissues such as the aorta. Recently the importance of the ADAMTS and ADAMTS-like glycoproteins in mediating pathological changes (which may be phenotypically indistinguishable from some mutations in the FBN1 gene) has been recognised. Members of this large family of proteins exhibit diverse and complex biological functions including both the inhibition and promotion of microfibril deposition and the mediation of focal adhesion formations. Finally it is clear that elastic fibre formation and function may be influenced enzymatically by the expression of transglutaminase, LOX and LOXL and that elastic fibres can exert considerable influence over tissue homeostasis via regulation of TGFβ and the BMP/GDF families of cytokines.

Fibrillin microfibril and elastic fibre biology is highly complex. This complexity presents the research community with a difficult technical challenge in unravelling the multiple molecular and cellular interactions. However, understanding the multiplicity of elastic fibre protein interactions also presents a clear future opportunity to intervene in, and to recognise, disease processes and mechanisms of ageing and to control tissue development for tissue engineering applications.
